# Mechanical Properties of Treadmill Surfaces Compared to Other Overground Sport Surfaces

**DOI:** 10.3390/s20143822

**Published:** 2020-07-09

**Authors:** Enrique Colino, Jose Luis Felipe, Bas Van Hooren, Leonor Gallardo, Kenneth Meijer, Alejandro Lucia, Jorge Lopez-Fernandez, Jorge Garcia-Unanue

**Affiliations:** 1 IGOID Research Group, Physical Activity and Sport Sciences Department, University of Castilla-La Mancha, 45071 Toledo, Spain; enrique.colino@uclm.es (E.C.); leonor.gallardo@uclm.es (L.G.); jorge.garciaunanue@uclm.es (J.G.-U.); 2 School of Sport Sciences, Universidad Europea de Madrid, 28670 Madrid, Spain; alejandro.lucia@universidadeuropea.es; 3 Department of Nutrition and Movement Sciences, NUTRIM School of Nutrition and Translational Research in Metabolism, Maastricht University, Universiteitssingel 50, 6229 ER Maastricht, The Netherlands; basvanhooren@hotmail.com (B.V.H.); kenneth.meijer@maastrichtuniversity.nl (K.M.); 4 Research Institute Hospital 12 de Octubre (‘imas12’), 28041 Madrid, Spain; lopezfej@uni.coventry.ac.uk; 5 Centre for Sport, Exercise and Life Science, Coventry University, Coventry CV1 5FB, UK

**Keywords:** sport surfaces, running, biomechanics, performance, injury risk, shock absorption, vertical deformation, energy restitution

## Abstract

The mechanical properties of the surfaces used for exercising can affect sports performance and injury risk. However, the mechanical properties of treadmill surfaces remain largely unknown. The aim of this study was, therefore, to assess the shock absorption (SA), vertical deformation (VD) and energy restitution (ER) of different treadmill models and to compare them with those of other sport surfaces. A total of 77 treadmills, 30 artificial turf pitches and 30 athletics tracks were assessed using an advanced artificial athlete device. Differences in the mechanical properties between the surfaces and treadmill models were evaluated using a repeated-measures ANOVA. The treadmills were found to exhibit the highest SA of all the surfaces (64.2 ± 2; *p* < 0.01; effect size (ES) = 0.96), while their VD (7.6 ± 1.3; *p* < 0.01; ES = 0.87) and ER (45 ± 11; *p* < 0.01; ES = 0.51) were between the VDs of the artificial turf and track. The SA (*p* < 0.01; ES = 0.69), VD (*p* < 0.01; ES = 0.90) and ER (*p* < 0.01; ES = 0.89) were also shown to differ between treadmill models. The differences between the treadmills commonly used in fitness centers were much lower than differences between the treadmills and track surfaces, but they were sometimes larger than the differences with artificial turf. The treadmills used in clinical practice and research were shown to exhibit widely varying mechanical properties. The results of this study demonstrate that the mechanical properties (SA, VD and ER) of treadmill surfaces differ significantly from those of overground sport surfaces such as artificial turf and athletics track surfaces but also asphalt or concrete. These different mechanical properties of treadmills may affect treadmill running performance, injury risk and the generalizability of research performed on treadmills to overground locomotion.

## 1. Introduction

Treadmills are widely used in different settings including sports training, exercise testing, rehabilitation and research [1]. Although it is frequently assumed that locomotion on a treadmill is a surrogate for ground locomotion, there is controversy as to the comparability of the biomechanical, physiological, perceptual or performance outcomes between the two conditions [1,2,3].

Insufficient familiarization and a lack of air resistance can make treadmill running differ from running overground [4,5,6]. However, there is recent meta-analytical evidence that differences can still be found between the two conditions independent of previous familiarization [3] and that the effect of air resistance becomes a significant confounder only at relatively high running speeds—approximately above 16 km/h, which is actually faster than the speeds used in most studies in the field [1]. Factors other than familiarization or air resistance might thus be involved. In this regard, the role of the belt dimensions and intra-belt speed fluctuations remains largely unclear but might be relatively small for modern treadmills with strong driving mechanisms that provide minimal intra-stride belt speed variability, including high-quality research-based treadmills [3]. On the other hand, the controversy in the field regarding the comparison of treadmill vs. overground running could also be caused by dissimilarities in the mechanical properties of the running surfaces used in the different studies [2,3,7,8]. Indeed, treadmills’ mechanical properties have an important influence—and in fact, greater than that of the lack of air resistance—on physiological responses [2,9] and can also affect running biomechanics [3], since athletes adjust their leg stiffness and dynamics when running on surfaces with different mechanical properties [10,11,12,13].

Although the mechanical properties of many sport surfaces (e.g., artificial turf pitches, athletics tracks, sports hall floors, tennis courts and gymnastic crash mats) are frequently assessed to ensure they meet the criteria established by sport international federations and other governing bodies [14], this is not the case for treadmill surfaces, for which there are yet no standardized criteria. In this sense, current regulations (both European and American) define constructive and general safety aspects without any mention of the mechanical properties of the surface [15,16,17]. The same limitation applies to the bulk of scientific research comparing treadmill and overground locomotion [3].

Assessing the mechanical properties of treadmill surfaces is therefore an important issue, not only in sports but also from a clinical perspective. Indeed, treadmill surfaces’ mechanical properties have a significant influence on peak plantar forces and metabolic energy consumption [8,18], and treadmill running has been associated with a lower risk of developing tibial stress fractures but an increased risk of overload injuries at the Achilles tendon compared to overground running [19,20,21], due to altered lower-extremity kinetics and kinematics.

Generally, regulations require that the three main mechanical properties of sports surfaces—shock absorption (SA), vertical deformation (VD) and energy restitution (ER)—are evaluated [22,23]. However, the few studies that have characterized treadmills’ mechanical properties in any way have mainly focused on surface stiffness [18,24]. Although stiffness is closely related to VD, it provides little information regarding SA and ER. In this context, and given that the mechanical properties of treadmills remain largely unknown, the main purpose of this study was to characterize SA, VD and ER among different treadmill models designed for fitness, research and rehabilitation purposes, and to compare the results with those obtained for other man-made surfaces typically used in sports—artificial turf and athletics track surfaces. In addition, the relationship between the different mechanical properties can provide a more comprehensive understanding of the behavior of the surface and its influence on athletes. Although these relationships have been previously studied in overground surfaces, they remain largely unknown for treadmills. Therefore, a second aim was to assess the relationship between SA, VD and ER and whether this relationship remained consistent across surfaces.

## 2. Materials and Methods

### 2.1. Sample

A total of 77 treadmills, 30 artificial turf pitches and 30 track and field tracks were included in the study. The treadmills comprised 70 conventional flat treadmills from fitness centers (fit-TR), 6 non-instrumented treadmills from different research laboratories (lab-TR), and one curved non-motorized treadmill (NM-TR) (Table 1). Artificial turf and track samples were selected randomly from a database of field tests performed by a certified laboratory.

### 2.2. Procedures

We assessed SA, VD, and ER with an advanced artificial athlete (AAA) device (Wireless Value; Emmen, The Netherlands) that consists of a mechanical drop test simulating the support of an athlete’s foot on the ground. The characteristics of the apparatus are thoroughly described in Section 12 of current FIFA standards [23], the model used here being a wireless handheld device that provided ease of operation and simple and fast measurements. Artificial turf and track surfaces were assessed at different locations in accordance with current FIFA and World Athletics protocols, respectively [23,25]. For that, we performed three repetitions of the drop test at each test location, with intervals of 30 ± 5 s. We discarded the results of the first test and calculated the mechanical properties of each location as the mean values of the second and third tests. The treadmills were assessed at three points as described elsewhere [26], performing only one drop test per location. For each surface included in the study, we calculated the SA, VD, and ER as the mean values of all the test locations.

### 2.3. Statistical Analysis

Data are presented as means and standard deviations (SDs). We used the Kolmogorov–Smirnov and Levene’s test to check the normality of the data distribution and homogeneity of variances, respectively. We compared mechanical properties across the three types of surfaces (fit-TR, artificial turf and athletics track) with a one-way analysis of variance (ANOVA) test, with the Bonferroni test used for post hoc pairwise comparisons. We used the same approach to compare the mechanical properties within the different fit-TR models. We calculated the effect size for the group effect (ES) with the partial Eta-squared (*η*_p_^2^) value with the following interpretation: small (*η*_p_^2^ = 0.01–0.059), medium (*η*_p_^2^ = 0.06– ≥ 0.14) and large effects (*η*_p_^2^ > 0.14). Finally, we also calculated the Pearson’s correlations between the three mechanical properties within each type of surface. We used the statistical software SPSS V24.0 for Windows and set the level of significance at *p* < 0.05.

## 3. Results

**Figure 1:**
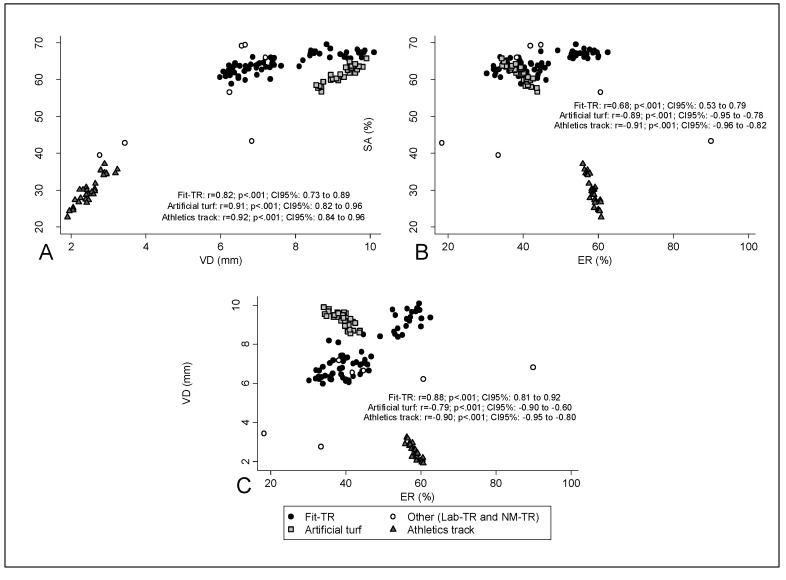
Correlations between mechanical properties within fit-TR, artificial turf and track surfaces (**A**: SA vs. VD; **B**: SA vs. ER; **C**: VD vs. ER). Abbreviations: ER, energy restitution; Fit-TR, treadmills from fitness centers; Lab-TR, laboratory treadmills; NM-TR, non-motorized treadmill; SA, shock absorption; VD, vertical deformation. Lab-TR were not included in the correlation analyses, as they did not follow the premises of normal distribution and homogeneity of variances.

We excluded lab-TR and NM-TR data from the analyses, as they did not follow the premises of normal distribution and homogeneity of variances. The results for these treadmills are shown for information in the graphical analysis (Figure 1).

When comparing the overall differences in the mechanical properties across the three types of surfaces (fit-TR, artificial turf, and track and field) we found a significant group (i.e., “type of surface”) effect for SA, VD and ER (Table 2). In post hoc pairwise comparisons, SA was lower in track than in the other two surfaces (*p* < 0.001 vs. both fit-TR and artificial turf) and lower in artificial turf than in fit-TR (*p* = 0.001). VD was also lower in track than in the other two surfaces (*p* < 0.001 vs. fit-TR and artificial turf, respectively) and lower in fit-TR than in artificial turf (*p* < 0.001). By contrast, ER was higher in track than in the other two surfaces (*p* < 0.001 vs. fit-TR and artificial turf) and also lower in artificial turf than in fit-TR (*p* = 0.002).

Table 3 shows the differences between the six fit-TR models, revealing a significant group effect for SA, VD and ER. The treadmill models of the brand Life Fitness (LF_97T_ and LF_DX_) displayed higher values of SA, VD and ER compared to the other treadmills (*p* < 0.01 for all cases), while the Precor model (PRE_956I_) showed the lowest values of VD and ER (*p* < 0.05 for all cases), with no significant differences in SA compared to the Technogym models.

Figure 1 shows the product-moment correlations between the mechanical properties of each surface, taking all of the fit-TR models as a single group. All the surfaces showed a strong positive correlation between the SA and VD, this association being slightly weaker for the fit-TR. As for the SA vs. ER and the VD vs. ER relationships, artificial turf and track surfaces showed a strong negative correlation in both cases, whereas positive correlations (moderate and strong, respectively) were found for fit-TR.

## 4. Discussion

Our results show differences between the mechanical properties of treadmill surfaces, artificial turf pitches and athletics tracks. Taken together, artificial turf surfaces comply with the international standards for both football [23] (SA, 55–70%; VD, 4–11 mm; ER, N/A) and rugby [27] (SA, 55–70%; VD, 5.5–11.0 mm; ER, 20–50%), and the track surfaces meet the criteria established by World Athletics when assessed with the AA [25] (SA, 35–50%; VD, 0.6–2.5 mm; ER: N/A). When compared to these surfaces, treadmills show statistically significant differences in all mechanical properties. Thus, treadmills have the highest SA ability of all the surfaces, while their VDs and ERs range between those of the artificial turf and the track, being much closer to the first. When compared to other surfaces such as asphalt or concrete—with SA values below 2%, and VDs and ERs close to 0 [7,28]—these differences are even higher. This suggests that, despite having been conceived for running and walking, the mechanical behavior of treadmill surfaces differs remarkably from that of other surfaces used for similar purposes such as tracks or asphalt roads. By contrast, treadmill surfaces seem to better reproduce the mechanical properties of the artificial turf.

Our results are in line with those of previous studies reporting that treadmill surfaces are usually more compliant than overground running surfaces [13] and also with those reporting that treadmill surfaces overall have a less compliant—here indicated by a lower VD—and higher damping behavior—here indicated by a higher ER—than artificial turf surfaces [9,29]. However, our findings regarding the mechanical behavior of treadmills cannot be generalized since there are large differences between treadmill models, even within the same brand. Indeed, our results show significant differences between the treadmills commonly used in fitness centers (fit-TR) of up to 6%, 3.1 mm and 25% in SA, VD and ER, respectively. These findings suggest that fit-TR may not be considered as homogeneous surfaces in terms of mechanical properties and that each treadmill model should be tested individually in order to characterize its mechanical behavior. Moreover, our results suggest that differences may exist between treadmill brands, as previously suggested [30], although the small sample of brands and models included in this study precludes the ability to draw general conclusions.

While keeping in mind that lab-TR could not be included in the statistical analyses, our results suggest that differences across lab-TR could be even greater than those reported for fit-TR. In this regard, some studies have shown that differences in the mechanical properties of treadmill surfaces can affect the metabolic cost and ground reaction forces during running [18,31], and others have reported that the varying mechanical properties of the running surface may result in premature fatigue or undesirable challenge during a certain task [32,33]. Collectively, these findings suggest that researchers, clinicians and athletes using a lab-TR for specific purposes must carefully choose the model to be used, since this may affect the generalizability of clinical assessments or research performed on the treadmill, potentially leading to erroneous research findings [3,13,18,31,34]. For example, our findings imply that marked differences in mechanical properties between treadmill and overground surfaces could critically affect footwear studies using treadmills to assess the effects of running shoes on running economy and running biomechanics [35,36,37], since the optimal footwear on a treadmill may not necessarily be the optimal footwear on an overground surface. Therefore, researchers using treadmills to reproduce overground conditions in research or clinical settings should attempt to use a treadmill whose surface mimics as closely as possible the mechanical properties of the specific overground surface, since the comparability between both conditions will vary depending on the treadmill platform [18]. We therefore encourage the persistent testing and reporting of the mechanical properties of the surfaces to allow reliable comparisons to be made in this context, especially in research that aims to investigate the relationship between treadmill and overground locomotion, or where there is the need to reproduce overground conditions for specific purposes—e.g., to investigate the effects of footwear.

Our results show a greater dispersion of treadmills’ mechanical properties compared to those of artificial turf and track surfaces (Figure 1). Our findings on the relationship between SA, VD and ER in artificial turf and track surfaces support previous studies reporting that an increased compliance (i.e., higher VD) in overground surfaces is associated with a reduction in the re-utilization of elastic energy (i.e., a lower ER) [38,39,40], which would lead to an increased metabolic cost and alterations in running kinematics. However, as opposed to overground surfaces, both SA and VD are directly proportional to ER in treadmills, meaning that treadmills with more shock-absorbing and compliant surfaces would increase energy return to the runners. This supports previous research pointing that the metabolic cost of running is greater for treadmills with stiffer running platforms [18,23], contrary to what is encountered overground [7]. Moreover, the fact that the ER of some lab-TR is drastically lower than that of track surfaces could also explain previous findings reporting that the metabolic cost at low [32] and submaximal speeds (with controlled air resistance) [2] is significantly higher on a treadmill compared to that on track surfaces. The increase in the treadmill ER as VD increases will most likely be due to the materials and structural components forming their surfaces, which determine their viscoelastic (or damping) properties relevant during the unloading phase. The latter may have relevant implications in terms of muscle activity and injury risk, as well as in terms of performance outcomes and the reproducibility of kinematic patterns when comparing treadmill to overground locomotion. In this sense, it has been reported that stiffer surfaces lead to increased muscle activity [41] and that surfaces providing increased mechanical cushioning affect running kinematics [11]. Nevertheless, the implications for performance and injury risk of surfaces with comparable stiffnesses but different damping properties remain unclear.

Overall, the present findings support the importance of regulating the mechanical properties of treadmill surfaces because (1) the mechanical properties of all sports surfaces are considered to be important determinants of performance and injury risk, and (2) our results indicate that the mechanical properties of treadmills vary across models and do not match those of other surfaces that are often used for walking and running. Moreover, since treadmills with very similar VD (which is an indicator of their stiffness) may differ strongly in SA and ER, our results also indicate that assessing and regulating only stiffness in treadmill surfaces may not suffice for fully characterizing their mechanical behavior. Similarly, relating research results to surface stiffness could potentially lead to misleading conclusions. Further research in this area may help manufacturers to design treadmills with surface properties that match those of specific overground surfaces, or treadmills with surface properties specifically designed to achieve certain purposes such as enhancing athletic performance or decreasing injury risk. Additionally, future research should assess whether mechanical properties of treadmill surfaces could correlate with other variables such as a treadmill’s usage time, temperature or kilometers traveled, which is something that the present research failed to investigate due to a lack of data.

## 5. Conclusions

The mechanical properties (shock absorption, vertical deformation and energy restitution) of treadmill surfaces differ significantly from those of commonly used overground sport surfaces such as artificial turf and athletics tracks. Our results also suggest that, unlike overground surfaces, treadmills with more shock-absorbing and compliant surfaces would be expected to increase energy return to the athletes. Moreover, our results show remarkable differences between different treadmill models, suggesting that treadmills will most likely vary in their comparability to overground surfaces depending on the mechanical properties of their platforms.

## Figures and Tables

**Table 1:** Characteristics of the treadmills included in the study.

Group	Brand	Model	Year of Manufacture	N	Code
**Fit-TR**	LifeFitness	Integrity Series 97T	2011	14	LF_97T_
	LifeFitness	Integrity Series DX	2019	9	LF_DX_
	Technogym	Jog 500	2012	5	TEC_Jog500_
	Technogym	Jog 700 Excite	2008	12	TEC_Jog700_
	Technogym	Runartis	2018	22	TEC_Runart_
	Precor	956i	2009	8	PRE_956i_
**Lab-TR**	Technogym	Excite-Med	2018	1	TEC_E-M_
	HP Cosmos	Pulsar lt 3P	2004	1	HP_Pul2004_
	HP Cosmos	Pulsar lt 3P	2013	1	HP_Pul2013_
	HP Cosmos	Saturn	2006	1	HP_Sat_
	HP Cosmos	Venus	2016	1	HP_Ven_
	Lode	Valiant 2 Rehab	2017	1	LOD_Rehab_
**NM-TR**	Technogym	Skillmill	2019	1	TEC_Skill_

Abbreviations: Fit-TR, treadmill from fitness centers; Lab-TR, laboratory treadmills; NM-TR, non-motorized treadmill.

**Table 2:** Mechanical properties of the main types of surfaces.

	Treadmill (Fit-TR)			Artificial Turf			Track			Group Effect (*p*-Value and ES)
**SA (%)**	64	±	2	62	±	2 ^Ŧ^	30	±	4 ^Ŧ.^*	*p* < 0.001, ES = 0.96
**VD (mm)**	7.6	±	1.3	9.3	±	0.4^Ŧ^	2.5	±	0.4 ^Ŧ.^*	*p* < 0.001, ES = 0.87
**ER (%)**	45	±	11	39	±	3^Ŧ^	58	±	1 ^Ŧ.^*	*p* < 0.001, ES = 0.51

Data are mean (±) SD. Abbreviations: ER, energy restitution; SA, shock absorption; VD, vertical deformation. Symbols: ^Ŧ^ *p* < 0.05 vs. treadmill; * *p* < 0.05 vs. artificial turf. Of note, World Athletics states that the artificial athlete (AA) device should be used instead of the advanced artificial athlete (AAA) to assess the mechanical properties of track surfaces. The equivalence between both test apparatus has been previously described [22]. Thus, the above reported values for track surfaces (which were obtained using the AAA) would be equivalent to SA and VD values of ≈35.5% and ≈ 1.73 mm, respectively, when assessed with the AA.

**Table 3:** Mechanical properties by model of treadmill.

	LF_97T_ (a)			LF_DX_ (b)			TEC_JOG500_ (c)			TEC_JOG700_ (d)			TEC_RUNART_ (e)			PRE_956I_ (f)			Group Effect (*p*-Value and ES)
**SA(%)**	67	±	1 ^c.d.e.f^	68	±	1 ^c.d.e.f^	62	±	2	63	±	2	64	±	2	63	±	2	*p* < 0.001, ES = 0.69
**VD(mm)**	9.6	±	0.3 ^b.c.d.e.f^	8.6	±	0.2 ^c.d.e.f^	7.2	±	0.4 ^f^	6.5	±	0.4 ^e^	7.0	±	0.6 ^f^	6.4	±	0.2	*p* < 0.001, ES = 0.90
**ER(%)**	58	±	3 ^b.c.d.e.f^	53	±	4 ^c.d.e.f^	44	±	2 ^d.f^	39	±	5^f^	40	±	2^f^	33	±	1	*p* < 0.001, ES = 0.89

Abbreviations: ER, energy restitution; SA, shock absorption; VD, vertical deformation. Symbols: a–f, *p* < 0.005 vs. (a), (b), (f), respectively.
